# How light and biomass density influence the reproduction of delayed *Saccharina latissima* gametophytes (Phaeophyceae)

**DOI:** 10.1111/jpy.12976

**Published:** 2020-02-28

**Authors:** Alexander Ebbing, Ronald Pierik, Tjeerd Bouma, Jacco C. Kromkamp, Klaas Timmermans

**Affiliations:** ^1^ Department of Estuarine and Delta Systems NIOZ Royal Netherlands Institute for Sea Research PO Box 140 4401 NT Yerseke The Netherlands; ^2^ Department Ocean Ecosystems University of Groningen PO Box 72 9700 AB Groningen The Netherlands; ^3^ Department of Biology Utrecht University Padualaan 8 3584 CH Utrecht The Netherlands

**Keywords:** Gametophyte, Initial Gametophyte Density, Interaction, Kelp, Lifecycle control, Light intensity, Light quality, Photosynthetically Usable Radiation, Reproduction, *Saccharina latissima*, Vegetative growth

## Abstract

Kelp life‐cycle transitions are complex and susceptible to various (a)biotic controls. Understanding the microscopic part of the kelp's lifecycle is of key importance, as gametophytes form a critical phase influencing, among others, the distributional limits of the species. Many environmental controls have been identified that affect kelp gametogenesis, whose interactive effects can be subtle and counterintuitive. Here we performed a fully factorial experiment on the (interactive) influences of light intensity, light quality, and the Initial Gametophyte Density (IGD) on *Saccharina latissima* reproduction and vegetative growth of delayed gametophytes. A total of 144 cultures were followed over a period of 21 d. The IGD was a key determinant for reproductive success, with increased IGDs (≥0.04 mg DW · mL ^−1^) practically halting reproduction. Interestingly, the effects of IGDs were not affected by nutrient availability, suggesting a resource‐independent effect of density on reproduction. The Photosynthetically Usable Radiation (PUR), overarching the quantitative contribution of both light intensity and light quality, correlated with both reproduction and vegetative growth. The PUR furthermore specifies that the contribution of light quality, as a lifecycle control, is a matter of absorbed photon flux instead of color signaling. We hypothesize that (i) the number of photons absorbed, independent of their specific wavelength, and (ii) IGD interactions, independent of nutrient availability, are major determinants of reproduction in *S. latissima* gametophytes. These insights help understand kelp gametophyte development and dispersal under natural conditions, while also aiding the control of in vitro gametophyte cultures.

**Abbreviations**
IGD: initial gametophyte density
PUR: photosynthetically usable radiation
ClO^−^: hypochlorite

Kelp species of the family *Laminariaceae* have a heteromorphic lifecycle that alternates between haploid gametophytes and diploid sporophytes. In contrast to the macroscopic sporophytes, the haploid gametophytes are of a microscopic nature and especially delayed gametophytes are relatively understudied (Bartsch et al. 2008). Delayed gametophytes remain vegetative under limiting conditions (Kinlan et al. 2003), disperse through fracturing (Destombe and Oppliger 2011), can persist for prolonged periods of time (Carney 2011), even up to years (Zhao et al. 2016), and remain highly sensitive to changes in environmental quality (Edwards 2000, Carney and Edwards 2006). The asexual reproduction, growth, and increase of gametophyte biomass is regarded to be the adaptive form for stressful environments (Dieck 1993). To date, the lifecycle controls that determine whether delayed gametophytes persist their asexual vegetative growth or rather start gametogenesis (i.e., sexual reproduction to form sporophytes) remains open for exploration. A better understanding on this microscopic part of the kelp's lifecycle is highly needed, as this phase largely determines their recruitment success (Wiencke et al. 2006, Fredersdorf et al. 2009). The transition to the generative phase is furthermore thought to be highly susceptible to environmental perturbations, and hence a critical process in determining the distributional limits of the species (Destombe and Oppliger 2011).

Whether kelp gametophytes initiate gametogenesis may be influenced by a range of abiotic factors such as temperature (Lüning and Neushul 1978, Morita et al. 2003), light intensity (Hsiao and Druehl 1971, Bolton and Levitt 1985), photoperiod (Hsiao and Druehl 1971, Choi et al. 2005), and nutrient availability (Harries 1932, Martins et al. 2017). Light intensity has been described as a generic abiotic factor controlling gametogenesis, with broad light intensity gradients in which gametogenesis was successfully induced (Lüning 1980, Lee and Brinkhuis 1988). Especially the spectral composition of light is considered a major influencer of gametogenesis, with blue light acting as a major inducer of gametogenesis (Lüning and Dring 1972, 1975, Ratcliff et al. 2017). The combination of light intensity and light quality can be functionally integrated as the Photosynthetically Usable Radiation (PUR; Fig. 1). PUR as an abiotic lifecycle control has never been assessed in kelp gametophytes. Integrating light intensity and light quality into PUR, as a single variable, further elaborates how light quality functions as a gametogenesis inducer, as PUR consists out of the light quality‐dependent photon flux of absorbed photons by an organism (Orefice et al. 2016).

**Figure 1:**
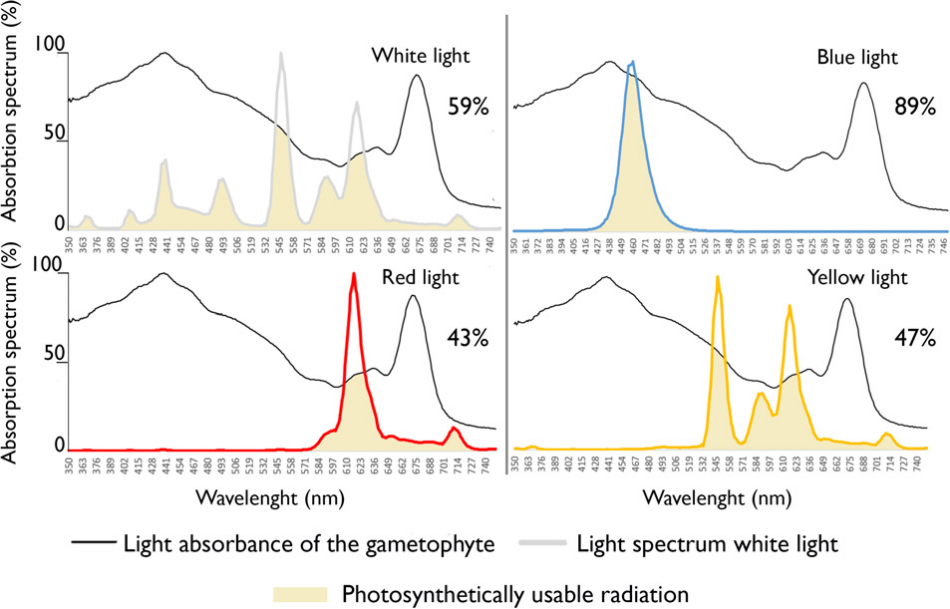
The light absorbance spectrum of *Saccharina latissima* gametophytes (black line) projected over the spectral distribution of four light qualities (white, yellow, red, and blue), produced by different experimental sources. Light was measured at different wavelengths from 400 nm until 700 nm, and peak emission strength was normalized to 1 and plotted against the absorbance of the culture (%).

Biotic factors have also been identified as potential lifecycle control mechanisms for gametogenesis, especially within the *Phaeophyceae* (Pohnert and Boland 2002, Frenkel et al. 2014). Most studies on the *Phaeophyceae* have focused on sexual pheromones like ectocarpene (Müller et al. 1971), fucoserratene (Müller and Jaenicke 1973), or lamoxirene (Marner et al. 1984). Culture density has been shown to influence reproduction, with higher densities resulting in lower reproductive success (Reed 1990, Reed et al. 1991, Choi et al. 2005, Carney and Edwards 2010). Culture density was hereby always described as an indirect biotic factor, with population size also affecting other primary abiotic factors like nutrient availability or light intensity. No studies have looked at gametophyte population density as a direct biotic factor regulating reproduction, independent of nutrient availability, or light intensity. Since density‐dependent behavioral mechanisms (e.g., quorum sensing) are found widespread within the eukaryotic kingdom (Amin et al. 2012), including the sporophytes of the Phaeophyceae (Dayton et al. 1984), such density‐dependent mechanisms might also affect gametophytes. In the case of gametophytes, population density (mg DW · mL^−1^) might be at the heart of whether gametophytes initiate gametogenesis or keep growing vegetatively.

Since the gametophyte life phase is considered to be the adaptive form for stressful environments, gametophyte vegetative growth may be expected to be promoted under sub‐optimal conditions (Lüning 1980). The Initial Gametophyte Density (IGD) may therefore have a substantial influence on whether a single gametophyte perceives its environment optimal or as sub‐optimal. If a higher IGD simulates suboptimal conditions it would especially influence the reproduction of delayed gametophytes, since prolonged periods of vegetative growth prior to gametogenesis automatically results in higher IGDs, therefore lowering reproductive success. Understanding the direct influence of IGD on delayed gametophytes is especially important for the seaweed industry, where genetic strain development is still considered a major challenge (Kim et al. 2017). Strain development in kelp is established using gametophyte clone cultures that have grown vegetatively for prolonged periods of time, hence resulting in artificially increased IGDs to levels that might be considered sub‐optimal for reproduction.

Light intensity, light quality, and their overarching abiotic factor (PUR), combined with the IGD as direct biotic lifecycle control, have to our knowledge never been investigated in a full factorial design for delayed gametophytes. Here we address the question on how the interaction of such environmental factors influences reproduction and the vegetative growth of delayed kelp gametophytes, using the economically important North Atlantic species *Saccharina latissima*. We hypothesize that lifecycle control drivers include (i) IGD as a direct biotic control, with higher gametophyte densities inhibiting reproduction, thus promoting vegetative growth; and (ii) PUR as an abiotic lifecycle control that functionally integrates the influence of both light quality and light intensity.

## Methods

### Saccharina latissima *sporophyte collection*

Ripe *Saccharina latissima* sori were collected along the coast of Flekkefjord, Norway (58.2983751, 6.1107353° E) on December 1, 2016. Ten parental individuals were pooled, where the ripe sori were cut out of the blade and cleaned thoroughly using absorbent paper. The sori were submerged in hypochlorite 0.15% (ClO^−^) and subsequently washed in pasteurized seawater (80°C for 5 h in three cycles). The cleaned sori were then placed in an incubator (12°C) overnight in order to dry. The next day the sori were placed in to flasks (400 mL) filled with pasteurized seawater for zoospores to be released, after which the zoospores developed into gametophytes through time. The gametophyte stock cultures were hereafter incubated at (12°C) under red light (30 μmol photons · m^−2^ · s^−1^; 12:12 h), using f/2 medium (Guillard and Ryther 1962). These cultures were incubated for 343 d prior to the start of the experiment in high‐density cultures (˃0.08 mg DW · mL^−1^). During this period the cultures grew vegetatively and were monitored and refreshed on a monthly basis.

### Light conditions

Randomly filled 24‐well plates (*n* = 36) with a volume of 3 mL were placed under five different light intensities (5, 10, 30, 60, and 80 μmol photons · m^−2^ · s^−1^) and four different light qualities (White‐, Blue‐, Red‐, and Yellow light; Fig. 1). The light qualities in this experiment were provided through either fluorescent tube lights (warm white) or LEDs. Tube lights were used for the colors white, red, and yellow. The colors red and yellow were achieved using specially designed color sleeves (Eurolite, Vadodara, India). It was impossible to achieve high irradiances of blue light using tube light sleeves, therefore we had to use blue LEDs in this experiment. We choose to use a different light intensity gradient for red light because we had no material at our disposal to increase the light intensity above 60 μmol photons · m^−2^ · s^−1^. Spectral distributions were measured using a modular multispectral radiometer (TriOs Ramses ARC, Rastede, Germany; Heuermann et al. 1999; Fig. 1). Variations in light intensity were achieved through specific placements of the cultures in respect to the light sources.

### Gametophyte culture measurements

Part of the stock gametophyte culture was diluted at the start of the experiment (Fig. S1 in the Supporting Information), to four Initial Gametophyte Densities (0.01, 0.02, 0.04, and 0.08 mg DW · mL^−1^). Fluorometry was used to estimate the biomass for IGD as well as further measurements through time, using the chlorophyll‐a concentration [Chl *a*] as a proxy for phytoplankton biomass (Huot et al. 2007). This was done by extrapolating measurements from a Chl‐*a* calibration line (Fig. S2 in the Supporting Information), coming from fluorometry measurements (Fast Ocean/Act2 FRRF, Chelsea Technologies Group Ltd) and relating this to freeze‐dried gametophyte dry weight (DW) measurements using 21 gametophyte cultures (60 mL). This extrapolation was necessary because of the very low quantities of gametophyte biomass in the 3 mL wells. The maximum PSII photosynthetic efficiency (*F*_v_/*F*_m_; Suggett et al. 2009), a proxy of cell viability, was furthermore measured using the FRRF and was followed during the experiment. The samples were dark‐adapted overnight before these measurements were taken (Fig. S3 in the Supporting Information).

### Reproductive success

Reproductive success, that is, number of successfully formed young sporophytes (≥25 μm length) per mL (Fig. S4 in the Supporting Information), was determined on day 21 (*cf* Choi et al. 2005, Martins et al. 2017). Microscopic observations showed that the young sporophytes only developed on the bottom of the well plates, and all were counted per triplet of the experimental conditions. After 21 d, all fertilized oogonia had developed into small sporophytes and the sizes of the sporophytes were still small enough for accurate counting of the single individuals.

### Photosynthetically usable radiation

A spectrophotometer (Agilent Cary 100 UV‐VIS fitted with a Labsphere DRA –CA‐3300 integrating sphere) was used to measure the absorbance spectrum of the gametophytes (Fig. 1). The absolute absorbed light per specific wavelength was then used for the calculation of PUR under the Photosynthetic Active Radiation spectrum (400–700 nm), using the following equation:

$$\sum_{k=400}^{700}\text{PAR}\left(\lambda \right)a\left(\lambda \right)d\lambda$$

where *a*(λ) is described as the probability that a photon of a given wavelength will be absorbed by the cells, which is derived from the absorption spectrum of gametophytes at the given wavelength (λ) and cell size (*d*; Orefice et al. 2016).

### Nutrient experiment

A nutrient experiment was conducted to investigate the effects of nutrient availability on reproduction, using identical experimental protocols as the full factorial experiment described above (12°C; 30 μmol photons · m^−2^ · s^−1^, white light). Cultures in this experiment were either placed in pasteurized seawater (nutrient poor) or in seawater enriched with f/2 medium (Guillard and Ryther 1962). The experiment was done using a dilution gradient of six IGDs (0.007, 0.012, 0.22, 0.038, 0.07, and 0.12 mg DW · mL^−1^). We plotted the relative reproductive success (sporophytes · mg^−1^) on the y‐axis instead of the reproductive success (sporophytes · mL^−1^), by calculating the amount of sporophytes that were produced per mg dry weight IGD instead of mL culture.

### Statistical analysis

All statistical analysis was done using SPSS 20.0.0 statistical package (SPSS Inc., Chicago, IL, USA) and Sigmaplot 13.0 (Systat software Inc., London, UK). A linear regression using a second‐order polynomial (parabola) was fit over both the effects of IGD and PUR on the reproductive success in R (R Core Team 2018) after log transformation of reproduction, IGD, and PUR values. This function was chosen through Akaike information criterion (AIC) model comparison of linear, parabolic, and log–log parabolic functions (Akaike 1969). Predictors for the vegetative growth were evaluated using a stepwise linear regression (fixed factors: light intensity, light quality, and IGD). All data were normally distributed and analyzed for homogeneity using the Levene's test of variance. In case of unequal variances, a robust test of equality of means for unequal variances was applied (Welch t‐test). A Games–Howell nonparametric post hoc comparison was subsequently applied to test for significant differences between the subgroups (light qualities, light intensities, Nutrients, and IGDs). If the data were found to be homogeneous a one‐way ANOVA was applied followed by the conservative Scheffe post hoc test to determine which factor level was responsible for the specific treatment differences. All tests were run with a significance level of 0.05%. Data of the reproductive success of the gametophytes (*n* = 102) and their vegetative growth (*n* = 144) are presented as mean ± SD. A Contour plot is also added on the bottom of the 3d scatterplot in order to increase the clarity of the data. These contour plots consist out of smoothed averages of the displayed z‐axis of the scatterplots (Loess smoother, sampling portion = 0.8, interval = 6).

## Results

### Saccharina latissima *reproductive success*

**Figure 2:**
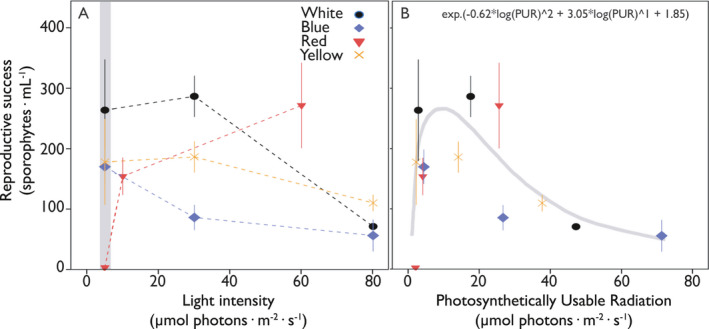
The influence of light intensity (μmol photons · m^−2^ · s^−1^) and PUR (μmol photons · m^−2^ · s^−1^) on the reproductive success of *Saccharina latissima* gametophytes using an IGD of 0.01 mg · mL ^−1^. The influence of light intensity (*x*‐axis, μmol photons · m^−2^ · s^−1^) and light quality (legend) on reproductive success (sporophytes · mL ^−1^) is depicted on side A, with the dotted lines representing the linear interpolation between the different data points. A gray bar is depicted on the left side in order to highlight the low light intensity environments described in the discussion. The influence of PUR (*x*‐axis, μmol photons · m^−2^ · s^−1^) on reproduction (sporophytes · mL ^−1^) is depicted on side B, with the color of the data points corresponding to the light qualities (legend). A regression (gray line) is fitted through these data points and the equation describing the regression is written in the upper right corner. Values are expressed as mean ± SD,* n* = 3.

**Figure 3:**
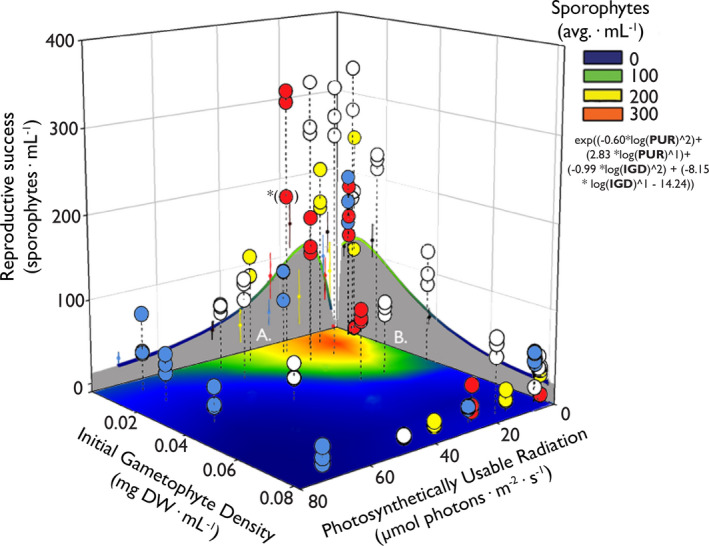
The interaction between IGD (*x*‐axis; mg DW · mL ^−1^) and PUR (*y*‐axis; μmol photons · m^−2^ · s^−1^) on the reproductive success of *Saccharina latissima* (*z*‐axis; sporophytes · mL ^−1^), with the colors of the dots representing the used light qualities. A regression is fitted over the 3d scatterplot and the corresponding equation can be found under the legend. The effects of the two separate lifecycle controls are depicted on the sides in the form of regressions, with PUR (gray surface, side A) or the IGD (gray surface, side B) as single variables. The interaction between PUR and the IGD as lifecycle controls is further clarified in the form of a smoothed contour plot. Colors match the colors in the legend. Error bars on the sides = ± SE,* n* = 102.

Reproduction was induced under different Initial Gametophyte Densities (IGD), light intensities, and light qualities (Figs. 2 and 3) and quantified as the number of sporophytes formed. Reproductive success (sporophytes · mL^−1^) became visible after 14 d, and was significantly influenced by all three environmental factors (Tables S1, S2, S3 in the Supporting Information), ranging from 336 sporophytes (white light; 5 μmol photons · m^−2^ · s^−1^; 0.02 mg DW · mL^−1^) to 1 sporophyte (red light; 5 μmol photons · m^−2^ · s^−1^; 0.093 mg DW · mL^−1^; Fig. 3). White light led to the highest reproductive success of all light qualities tested under optimal IGD conditions (0.01 mg · mL^−1^), whereas cultures in blue light had the lowest reproductive success, especially at higher light intensities (Fig. 2a). Cultures placed under yellow and red light gave, apart from the clear absence of reproduction under low red light conditions (5 μmol photons · m^−2^ · s^−1^), average results in terms of reproduction (Fig. 2a). High light intensities (≥80 μmol photons · m^−2^ · s^−1^) resulted in significantly lower reproduction under all light qualities (Table S3). The inhibitory effect of high light intensities on reproduction became more pronounced when plotting reproductive success against PUR. This analysis reveals systematically lower reproduction at a calculated PUR exceeding 26.8 μmol photons · m^−2^ · s^−1^, independent of light quality (Fig. 2B). Importantly, the PUR range is built up from a variety of light intensities and light qualities, accurately predicting reproduction irrespective of how specific PUR values were composed (regression in Fig. 2b; Table S4 in the Supporting Information).

Reproduction is influenced positively as well as negatively by the combination of IGD and PUR, resulting in an interaction of these two factors determining an IGD optimum between 0.02 and 0.01 mg DW · mL^−1^ and a PUR optimum between 14.2 μmol and 25.7 μmol photons · m^−2^ · s^−1^ (i.e., see 2d scatterplots A & B of Fig. 3). There was furthermore a pronounced decrease in reproductive success when PUR went above 26.8 μmol photons · m^−2^ · s^−1^, regardless of IGD. The regression describing the influence of IGD and PUR on the reproductive success was fitted (Table S5 in the Supporting Information; Linear regression: *F*_4,97_ = 40.88, *R* ^2^ = 0.628, *P* < 0.001). The representation of the interaction between IGD and PUR on the reproduction of *Saccharina latissima* is shown as a contour plot on the bottom of Figure 3. Note that the interactive effects of both the IGD and PUR (contour plot) resulted in higher average reproductive optimums than represented by the regressions on the sides. At (*) for example, at an IGD of 0.01 mg DW · mL^−1^ interacting with a PUR of 26 μmol photons · m^−2^ · s^−1^ red light a reproductive success of 190 sporophytes · mL^−1^ was observed, which is higher than what is calculated in both regressions.

**Figure 4:**
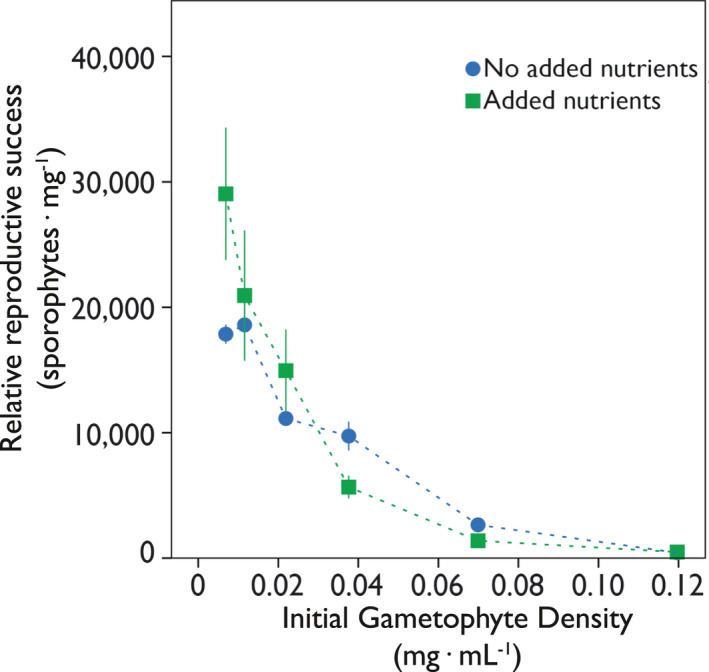
The influence of IGD (mg DW · mL ^−1^) on the relative reproductive success (sporophytes · mg^−1^) of *Saccharina latissima* gametophytes. The relative reproductive success of cultures with f/2 medium (added nutrients) and cultures in regular seawater (no added nutrients) are depicted as mean ± SD; n = 3

Reproduction was also followed to investigate the role of nutrients in interaction with the IGD as a direct influence on reproduction. Both pasteurized seawater (no added nutrients) as well as the f/2 medium (added nutrients) showed similar rates of reproduction (Fig. 4; Table S6 in the Supporting Information; ANOVA: *F*_1,35_ = 0.047, *P* ≥ 0.05), with decreasing IGDs resulting in increased levels of reproduction, independent of nutrient availability. Only at the lowest IGD (0.007 mg · mL^−1^) did the cultures without added nutrients show a decrease in relative sporophyte density. Although the observed reproduction was very similar between the treatments, the sizes of the individual sporophytes differed visually, with the treatments with added nutrients containing larger sporophytes. This last observation is purely anecdotal, since we did not quantitatively measure sporophyte size during this experiment.

### Vegetative growth

**Figure 5:**
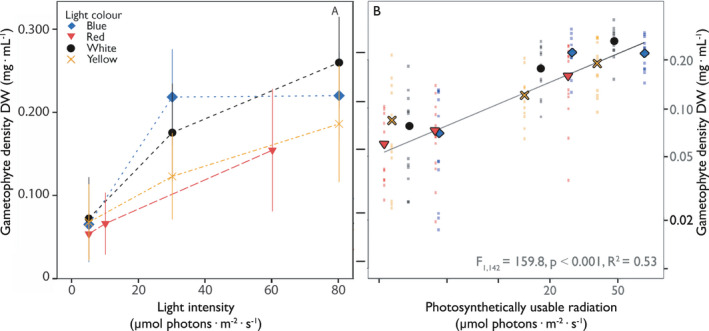
The influence of light intensity and the PUR on the vegetative growth of *Saccharina latissima* gametophytes. Gametophyte biomass (mg DW · mL ^−1^) on day 21 under different light intensities (μmol photons · m^−2^ · s^−1^) following different light qualities (legend) is depicted in A, with the dotted line representing the linear interpolation between the different data points. Side B depicts the gametophyte biomass (mg DW · mL ^−1^) on day 21 under different PURs (μmol photons · m^−2^ · s^−1^) following different light qualities (legend), with the line representing the correlation between the different data points (R^2^ is depicted in the lower right side). Note that both axes of B. are on a log scale. Values in A. are expressed as mean ± SD, while values in B. are expressed as a scatterplot with the mean (symbols), n = 12.

Gametophytes grew vegetatively in all cultures under all experimental conditions (Fig. 5). Primary predictor for the vegetative biomass accumulation in Figure 5a was light intensity (*R* ^2^ = 0.477), followed by IGD (R^2 ^= 0.235) and subsequently light quality (*R* ^2^ = 0.054; Tables S7 and S8 in the Supporting Information). Low light intensities (<30 μmol photons · m^−2^ · s^−1^) reduced the vegetative growth the most (Table S9 in the Supporting Information; Welch ANOVA: *F*_4,41_ = 50.37, *P* < 0.05), with significantly lower biomass found when grown at 10 and 5 μmol photons · m^−2^ · s^−1^ (Games–Howell, *P* < 0.05). Gametophytes grew significantly more at 30 μmol photons · m^−2^ · s^−1^, after which biomass accumulation of the gametophytes leveled off with only slight further increases in biomass at 80 μmol photons · m^−2^ · s^−1^. While light quality under comparable light intensities had limited influence on the vegetative growth of *Saccharina latissima* gametophytes (Fig. 5; Table S10 in the Supporting Information; ANOVA: *F*_2,105_ = 2.970, *P* ≥ 0.05) some distinctions can be made. The highest growth was achieved under white light 80 μmol photons · m^−2^ · s^−1^, whereas growth under blue light already started to plateau at 30 μmol photons · m^−2^ · s^−1^, independent of IGD (Fig. S5 in the Supporting Information). PUR as abiotic factor (Fig. 5B) was also plotted against the observed vegetative growth, with a resulting correlation of *R* ^2^ = 0.53, irrespective of the light quality used. The large spread of the data points in the scatterplot is, among other things, a result of grouping the different IGDs. Plotting the IGDs separately resulted in higher correlations between PUR and vegetative growth for all light qualities, apart from cultures places under blue light (Fig. S6 in the Supporting Information).

## Discussion

### Initial Gametophyte Density (IGD) as a direct biotic lifecycle control

This study presents the results for the effects of the (a)biotic factors (i) IGD, (ii) light intensity, (iii) light quality, and the overarching *iv)* PUR on reproduction and the vegetative growth of delayed *Saccharina latissima* gametophytes during a 21 d experimental period. Reproduction became visible after 14 d in treatment, coinciding with periods found in other studies with Laminariaceae (Morelissen et al. 2013, Ratcliff et al. 2017). Reproduction decreased with increasing IGDs, under all light intensities and light qualities. These results are in agreement with data obtained by Choi et al. (2005) and Reed (1990), Reed et al. (1991), where increasing spore densities of *Undaria pinnatifida* and *Macrocystis pyrifera* resulted in lower sporophyte counts. Carney and Edwards (2010) found similar negative correlations between reproduction and culture density of non‐delayed *Macrocystis pyrifera* gametophytes. Interestingly, these authors also studied delayed gametophytes (88 d), and found no significant difference in reproduction in three of their four starting zoospore densities. Although their study reported gametophyte density as the number of gametophytes per area, rather than gametophyte biomass per volume as used here, similar trends could be observed between our highest starting densities. Indeed, their highest density treatment of 212 gametophytes · mm^−2^ (±15.3) showed a significant decrease in reproduction, comparable to what we observed in our higher IGD samples (≥0.04 mg · mL^−1^) of *S. latissima* gametophytes.

The experimental data support our hypothesis that density has a direct influence on *Saccharina latissima* reproduction, with high IGDs (>0.04 mg · mL^−1^) practically halting reproduction. Nutrient addition had no significant influence on reproduction and the reproductive success did not follow the observed differences in *F*_v_/*F*_m_ ratio, a proxy for cell viability (Suggett et al. 2009; Fig. S3). These data demonstrate that the negative effects of gametophyte density on reproduction are not likely occurring via putative density‐associated nutrient deficiency. Self‐shading (i.e., light‐dependent effects) can also be ruled out because of the low culture densities, top‐down light placement of the light source and homogeneity of the cultures. Our results showed furthermore that light limitation did not negatively influence reproduction, apart from cultures placed under 5 μmol photons · m^−2^ · s^−1^ red light. This is in agreement with results by Lee and Brinkhuis (1988), who found no decrease in the reproductive success of female gametophytes under low light conditions (6 μmol photons · m^−2^ · s^−1^ white light). The exact mode of action of IGD as a direct biotic factor remains to be investigated. Whether the observed density‐dependent behavior is controlled pheromonally or is more similar to the autoinducers found in quorum sensing bacterial communities, is not yet known. It might even be possible that density‐dependent reproduction is the result of interkingdom signaling between gametophytes and bacteria, a phenomenon already studied within diatom communities (Amin et al. 2012).

Multiple hormones related to reproduction (i.e., ectocarpene, lamoxirene, and fucoserratene) have already been described for kelp gametophytes (Müller et al. 1971, Müller and Jaenicke 1973, Marner et al. 1984), making it feasible that one of these previously mentioned or novel compounds secreted by the gametophytes can accumulate in high‐density cultures, thereby suppressing reproduction. Suppressing reproduction under higher gametophyte densities could have benefits for their offspring, as the inverse correlation between IGD and reproductive success would prevent any future competition between sporophytes living in high‐density populations due to their competition for space (Dayton et al. 1984). The vegetative growth and the subsequent fragmentation of gametophyte branches therefore becomes the alternative option for dispersal (Destombe and Oppliger 2011). Moreover, in vitro work looking into reproduction, or gametogenesis in general, should take into account the IGD as a relevant biotic lifecycle control, especially regarding delayed gametophytes. The older a delayed gametophyte is, the more time it had to grow vegetatively, and the longer their vegetative growth period was, the higher the IGD automatically becomes, suppressing reproduction.

### Photosynthetically usable radiation as abiotic lifecycle control

There were interactions between light intensity and quality in determining reproduction, calling for a proxy that integrates both: PUR. Indeed, PUR seems to regulate reproduction in our delayed gametophyte cultures very tightly. Previous studies on light quality as a lifecycle control did not incorporate PUR (Lüning and Dring 1972, 1975, Ratcliff et al. 2017), so a comparison is complicated, especially because gametophyte densities were not quantified in the same way as done here. It is likely that gametophyte densities in the previously mentioned studies were in the lower range of the ones used here, as either the gametophytes were countable (Lüning and Dring 1972, 1975), or the cultures were diluted substantially into larger volumes of seawater (Ratcliff et al. 2017). Moreover, the light intensities reported were in the lower range of what we used here (6–15 µmol photons · m^−2^ · s^−1^). Interestingly, zooming into the low light intensities, low IGD region in Figure 2a (gray bar) reveals that a low intensity of red light resulted in very poor reproduction, whereas a similarly low intensity of blue light gave clear reproduction. This is entirely consistent with literature findings, such as by Lüning and Dring (1972). However, these conclusions shift when higher light intensities were used. Using higher light intensities of red light resulted in higher reproductive success and suggests that not so much light quality but the absorbed photon flux (PUR), irrespective of their wavelength, appears to be the important determinant regulator of reproduction. Importantly, when gametophyte densities become very high, the density effects overrule the effects of PUR and suppresses reproduction altogether.

Light intensity by itself was a strong predictor for the vegetative growth of gametophytes, with optima at 80 μmol photons · m^−2^ · s^−1^, under all light qualities and IGDs. Interestingly, biomass growth started to level off between 30 μmol photons m^−2^ s^−1^ and 80 μmol photons · m^−2^ · s^−1^. This corroborates with results of other studies, finding no effects on growth in gametophytes at irradiances of 30 μmol photons · m^−2^ · s^−1^ or higher (Lüning and Neushul 1978, Izquierdo et al. 2002, Choi et al. 2005). The influence of light quality was more limited, where its role on the vegetative growth is better explained through the usage of PUR as a parameter. Average gametophyte density (mg DW · mL^−1^) on day 21 correlated well with PUR (*R* ^2^ = 0.53), especially considering the interactive effects that were still present due to the different IGDs used. The correlation between vegetative growth and PUR, independent of light quality, becomes especially apparent when the interactive effects of IGD are taken out of the equation (Fig. S4). In this case, overall higher correlations were found under all light qualities except for cultures incubated under blue light, showing consistently lower correlations. The lower correlation under blue light is likely due to the plateauing biomass growth of cultures grown at a PUR of 71.4 μmol photons · m^−2^ s^−1^, irrespective of IGD. These high light intensities of blue light subsequently lowered the maximum quantum yield of the PSII substantially (Fig. S3), suggesting that photo inhibition was taking place (Gevaert et al. 2002).

To our knowledge, the gametophyte dry weight (mg · mL^−1^) of these small cultures (3 mL), has never been followed through time before. Using these small cultures was necessary for the feasibility of this full factorial experiment of such a large sample size. This makes it difficult, if not impossible, to compare our vegetative growth rates with cultures grown in similar condition. Furthermore, most research into the vegetative growth of gametophytes followed the surface area, the number of cells, or the length of gametophytes (Bolton and Levitt 1985, Carney and Edwards 2010, Morelissen et al. 2013, Martins et al. 2017). Ratcliff et al. (2017) used similar parameters to ours, looking at much larger volumes of gametophyte biomass dry weight (g · L^−1^), and found similar growth rates under comparable light conditions, also using f/2 medium. The difficulty of quantitatively comparing our results to other data is showing the need for concise and comparable methods of following gametophyte biomass in future studies.

Future work on the lifecycle controls in kelp will benefit from the inclusion of IGD and PUR in interaction with other lifecycle controls (e.g., temperature, day length, or other (a)biotic factors). The interaction between these lifecycle controls are also interesting from a more applied perspective, where finding the reproductive optimum can result in better production cost estimates and lower production costs. Advancements that are crucial in order to make large‐scale seaweed aquaculture economically feasible (van den Burg et al. 2016)

## Conclusions

Although there are clear interactive effects, two individual factors were identified as the most important determinants of reproduction and vegetative growth. The Initial Gametophyte Density was shown to be a dominant biotic factor influencing reproduction, outweighing light intensity or light quality. The Photosynthetically Usable Radiation, indicating the absorbed photon flux through the integration of both light intensity and light quality, is a second dominant (abiotic) determinant explaining the results on reproduction and the vegetative growth of kelp gametophytes. Light quality appears to act primarily through the efficiency in photon absorbance, as calculated through PUR. Light quality has hereby shown to be an abiotic factor that should be interpreted quantitatively instead of qualitatively as a color signal.

The authors want to thank Hortimare BV and their staff for the use of their *Saccharina latissima* gametophyte cultures. We furthermore want to thank Greg Fivash for his help and patients in the challenging journey of full factorial data analysis. Lastly, we want to thank the reviewers and editor for their constructive criticism, increasing the quality of the manuscript.

## Supporting information


**Figure S1.** Calibration curve between the chlorophyll a concentration (mg Chl · m^−3^), and *Saccharina latissima* gametophyte dry weight per mL (mg DW · mL^−1^). Gametophyte dry weights are extrapolations from 60 mL cultures, whose [Chl] concentration were measured using a FRRF fluorometer. The linear regression and correlation coefficient were *y* = 7E‐05*x* − 9E‐05 and 0.975 respectively.

**Figure S2.** The interaction between light intensity (μmol photons · m^−2^ · s^−1^_)_ and the light quality (white, blue, red, and yellow) on *Saccharina latissima* gametophyte biomass (mg DW · mL^−1^) of cultures starting with the Initial Gametophyte Density of 0.01 mg DW · mL^−1^. Biomass was measured on day 21 and the error bars are ± SE, *n* = 36.

**Figure S3.** The 3D scatterplot showing the interaction between the Fv/Fm, the IGD (mg · mL^−1^), and light intensity (μmol photons · m^−2^ · s^−1^) of *Saccharina latissima* gametophyte cultures grown under four different light qualities. The color of the dots correspond with the legend (white, blue, red, and yellow), thus corresponding with the *F*_v_/*F*_m_ value of the sample *n* = 144.

**Figure S4.** Scatterplots depicting the *Saccharina latissima* gametophyte biomass measured on day 21 (*y*‐axis) under different levels of Photosynthetically Usable Radiation (μmol photons · m^−2^ s^−1^). Four different light qualities (white, blue, red, and yellow) were used to grow out gametophyte cultures starting with four different Initial Gametophyte Densities (0.01, 0.02, 0.04, and 0.08 mg DW · mL^−1^). Values are “as is,” *n* = 36.

**Figure S5.** A photo of the starting culture in a well plate (IGD = 0.01 mg DW · mL^−1^).

**Figure S6.** A photo of a culture on day 21 (IGD = 0.01 mg DW · mL^−1^, 30 μmol · m^−2^ · s^−1^, white light). Sporophytes only formed on the bottom with gametophyte biomass being a bit blurry since it grew upward toward the light, out of focus.

**Table S1.** Predictors for the regression describing the correlation of the IGD and PUR on the reproduction of *Saccharina latissma* gametophytes in Figure 3 (*n* = 102). Included is the *R* ^2^ of the primary (PUR) and secondary (IGD) predictor combined.

**Table S2.** Predictors for the regression describing the correlation of PUR and the reproduction of *Saccharina latissima* gametophytes in Figure 2, using an IGD of 0.01 mg · mL^−1^.

**Table S3.** Games–Howell post hoc analysis for the influence of light quality on gametogenesis after we found significant differences using the robust test of variance. The mean difference is significant at *P* < 0.05.

**Table S4.** Games–Howell post hoc analysis for the influence of the IGD on gametogenesis after we found significant differences using the robust test of variance. The mean difference is significant at *P* < 0.05.

**Table S5.** Games–Howell post hoc analysis for the influence of light intensity on gametogenesis after we found significant differences using the robust test of variance. The mean difference is significant at *P* < 0.05.

**Table S6.** Robust test of variance for the effects of nutrients on the gametogenesis of *Saccharina latissima* gametophytes (Fig. 4; Welch and Brown‐Forsythe), after not passing the test of homogeneity of variances.

**Table S7.** Stepwise linear regression for the correlation between the gametophyte biomass on day 21 (mg DW · mL^−1^), the IGD, light intensity, and light quality (*n* = 144).

**Table S8.** Predictors that significantly influence gametophyte growth. Included is the *R* ^2^ of the primary (IGD) and secondary predictor (light intensity) combined.

**Table S9.** Games–Howell post hoc analysis for the influence of light intensity on the growth of gametophyte biomass (chlorophyll‐*a* concentration) on day 21 after we found significant differences using the robust test of variance. The mean difference is significant at *P* < 0.05.

**Table S10.** Scheffe post hoc analysis for the influence of the different IGDs on the growth of gametophyte biomass (chlorophyll‐*a* concentration) on day 21 after we found significant differences using a one‐way ANOVA. The mean difference is significant at *P* < 0.05.

## References

[jpy12976-bib-0001] Akaike, H. 1969 Fitting autoregressive models for prediction. Ann. I. Stat. Math. 21:243–7.

[jpy12976-bib-0002] Amin, S. A. , Parker, M. S. & Armbrust, E. V. 2012 Interactions between diatoms and bacteria. Microbiol. Mol. Biol. Rev. 76:667–84. 22933565 10.1128/MMBR.00007-12 PMC3429620

[jpy12976-bib-0003] Bartsch, I. , Wiencke, C. , Bischof, K. , Buchholz, C. M. , Buck, B. H. , Eggert, A. , Feuerpfeil, P. et al. 2008 The genus *Laminaria* sensu lato: recent insights and developments. Eur. J. Phycol. 43:1–86.

[jpy12976-bib-0004] Bolton, J. J. & Levitt, G. J. 1985 Light and temperature requirements for growth and reproduction in gametophytes of *Ecklonia maxima* (Alariaceae: Laminariales). Mar. Biol. 87:131–5.

[jpy12976-bib-0005] Carney, L. T. 2011 A multispecies laboratory assessment of rapid sporophyte recruitment from delayed kelp gametophytes. J. Phycol. 47:244–51. 27021856 10.1111/j.1529-8817.2011.00957.x

[jpy12976-bib-0006] Carney, L. T. & Edwards, M. S. 2006 Cryptic processes in the sea : A review of delayed development in the microscopic life stages of marine macroalgae. Algae 21:161–8.

[jpy12976-bib-0007] Carney, L. T. & Edwards, M. S. 2010 Role of nutrient fluctuations and delayed development in gametophyte reproduction by *Macrocystis pyrifera* (Phaeophyceae) in southern California. J. Phycol. 46:987–96.

[jpy12976-bib-0008] Choi, H. G. , Kim, Y. S. , Lee, S. J. , Park, E. J. & Nam, K. 2005 Effects of daylength, irradiance and settlement density on the growth and reproduction of *Undaria pinnatifida* gametophytes. J. App. Phycol. 17:423–30.

[jpy12976-bib-0009] Dayton, P. K. , Currie, V. & Gerrodette, T. I. M. 1984 Patch dynamics and stability of some California kelp communities. Ecol. Monogr. 54:253–89.

[jpy12976-bib-0010] Destombe, C. & Oppliger, L. V. 2011 Male gametophyte fragmentation in *Laminaria digitata*: A life history strategy to enhance reproductive success. Cah. Biol. Mar. 52:385–94.

[jpy12976-bib-0011] Dieck, I. T. 1993 Temperature tolerance and survival in darkness of kelp gametophytes (Laminariales, Phaeophyta) ‐ Ecological and biogeographical implications. Mar. Ecol. Prog. Ser. 100:253–64.

[jpy12976-bib-0012] Edwards, M. S. 2000 The Role of alternative life‐history stages of a marine macroalage: a seed bank analogue? Ecology 81:2404–15.

[jpy12976-bib-0013] Fredersdorf, J. , Müller, R. , Becker, S. , Wiencke, C. & Bischof, K. 2009 Interactive effects of radiation, temperature and salinity on different life history stages of the Arctic kelp *Alaria esculenta* (Phaeophyceae). Oecologia 160:483–92. 19330357 10.1007/s00442-009-1326-9

[jpy12976-bib-0014] Frenkel, J. , Vyverman, W. & Pohnert, G. 2014 Pheromone signaling during sexual reproduction in algae. Plant J. 79:632–44. 24597605 10.1111/tpj.12496

[jpy12976-bib-0015] Gevaert, F. , Creach, A. , Davoult, D. , Holl, C. , Seuront, L. & Lemoine, Y. 2002 Photo‐inhibition and seasonal photosynthetic performance of the seaweed *Laminaria saccharina* during a simulated tidal cycle: chlorophyll fluorescence measurements and pigment analysis. Plant, Cell Environ. 25:859–72.

[jpy12976-bib-0016] Guillard, R. R. L. & Ryther, J. H. 1962 Studies of marine planktonic diatoms. I *Cyclotella nana* Hustedt and *Detonula confervacea* (Cleve). Can. J. Microbiol. 8:229–39. 13902807 10.1139/m62-029

[jpy12976-bib-0017] Harries, R. 1932 An investigation by cultural methods of some of the factors influencing the development of the gametophytes and the early stages of the sporophytes of *Laminaria digitata*,* L. saccharina,* and* L. cloustoni* . Ann. Bot. 46:893–928.

[jpy12976-bib-0501] Heuermann, R. , Reuter, R. & Willkomm, R. 1999 RAMSES: a modular multispectral radiometer for light measurements in the UV and VIS. Environmental Sensing and Applications. 3821(1):279–85. 10.1117/12.364189

[jpy12976-bib-0018] Hsiao, S. I. C. & Druehl, L. D. 1971 Environmental control of gametogenesis in *Laminaria saccharina*. I. The effects of light and culture media. Can. J. Bot. 49:1503–8.

[jpy12976-bib-0019] Huot, Y. , Babin, M. , Bruyant, F. , Grob, C. , Twardowski, T. H. & Claustre, H. 2007 Does chlorophyll a provide the best index of phytoplankton biomass for primary productivity studies? Biogeosci. Disc. 4:707–45.

[jpy12976-bib-0020] Izquierdo, J. L. , Pérez‐Ruzafa, I. M. & Gallardo, T. 2002 Effect of temperature and photon fluence rate on gametophytes and young sporophytes of *Laminaria ochroleuca* Pylaie. Helgoland Mar. Res. 55:285–92.

[jpy12976-bib-0021] Kim, J. K. , Yarish, C. , Hwang, E. K. , Park, M. & Kim, Y. 2017 Seaweed aquaculture: cultivation technologies, challenges and its ecosystem services. Algae 32:1–13.

[jpy12976-bib-0022] Kinlan, B. P. , Graham, H. G. , Sala, E. & Dayton, P. K. 2003 Arrested development of giant kelp (*Macrocystis pyrifera*, Phaeophyceae) embryonic sporophytes: a mechanisms for delayed recruitment in perennial kelps? J. Phycol. 39:47–57.

[jpy12976-bib-0023] Lee, J. A. & Brinkhuis, B. H. 1988 Seasonal light and temperature interaction effects on development of *Laminaria saccharina* (Phaeophyta) gametophytes and juvenile sporophytes. J. Phycol. 24:181–91.

[jpy12976-bib-0024] Lüning, K. 1980 Critical levels of light and temperatures regulating the gametogenesis of three *Laminaria* species (Phaeophyceae). J. Phycol. 16:1–15.

[jpy12976-bib-0025] Lüning, K. & Dring, M. J. 1972 Reproduction induced by blue light in female gametophytes of *Laminaria saccharina* . Planta 104:252–6. 24481738 10.1007/BF00387080

[jpy12976-bib-0026] Lüning, K. & Dring, M. J. 1975 Reproduction, growth and photosynthesis of gametophytes of *Laminaria saccharina* grown in blue and red light. Mar. Biol. 29:195–200.

[jpy12976-bib-0027] Lüning, K. & Neushul, M. 1978 Light and temperature demands for growth and reproduction of laminarian gametophytes in southern and central California. Mar. Biol. 45:297–309.

[jpy12976-bib-0028] Martins, N. , Tanttu, H. , Pearson, G. A. , Serrão, E. A. & Bartsch, I. 2017 Interactions of daylength, temperature and nutrients affect thresholds for life stage transitions in the kelp *Laminaria digitata* (Phaeophyceae). Bot. Mar. 60:109–21.

[jpy12976-bib-0502] Marner, F. J. , Müller, B. & Jaenicke, L. 1984 Lamoxirene structural proof of the spermatozoid releasing and attracting pheromone of Laminariales. Zeitschrift für Naturforschung 39(1):689‐91. 10.1515/znc-1984-0629

[jpy12976-bib-0029] Morelissen, B. , Dudley, B. D. , Geange, S. W. & Philips, E. 2013 Gametophyte reproduction and development of *Undaria pinnatifida* under varied nutrient and irradiance conditions. J. Exp. Mar. Biol. Ecol. 448:197–206.

[jpy12976-bib-0030] Morita, T. , Kurashima, A. & Maegawa, M. 2003 Temperature requirements for the growth of young sporophytes of *Undaria pinnatifida* and *Undaria undarioides* (Laminariales, Phaeophyceae). Phycol. Res. 51:266–70.

[jpy12976-bib-0031] Müller, D. G. , Gassmann, G. & Lüning, K. 1979 Isolation of a spermatozoid‐releasing and ‐attracting substance from female gametophytes of *Laminaria digitata* . Nature 279:430–1. 16068180 10.1038/279430a0

[jpy12976-bib-0032] Müller, D. G. & Jaenicke, L. 1973 Fucoserraten, the female sex attractant of *Fucus serratus* L. (Pheaophyta). FEBS Lett. 30:137–9. 11947078 10.1016/0014-5793(73)80636-2

[jpy12976-bib-0033] Müller, D. G. , Jaenicke, L. , Donike, M. & Akintobi, T. 1971 Sex attractant in a brown alga: chemical structure. Science 27:743–55. 10.1126/science.171.3976.1132 17777600

[jpy12976-bib-0034] Orefice, I. , Chandrasekaran, R. , Smerilli, A. , Corato, F. , Caruso, T. , Casillo, A. , Corsaro, M. M. , Dal Piaz, F. , Ruban, A. & Brunet, C. 2016 Light‐induced changes in the photosynthetic physiology and biochemistry in the diatom *Skeletonema marinoi* . Algal Res. 17:1–13.

[jpy12976-bib-0035] Pohnert, G. & Boland, W. 2002 The oxylipin chemistry of attraction and defense in brown algae and diatoms. Nat. Prod. Rep. 19:108–22. 11902438 10.1039/a806888g

[jpy12976-bib-0036] R Core Team . 2018 R: A Language and Environment for Statistical Computing. Vienna, Austria Available at: https://www.r-project.org/

[jpy12976-bib-0037] Ratcliff, J. J. , Soler‐Vila, A. , Hanniffy, D. , Johnson, M. P. & Edwards, M. D. 2017 Optimisation of kelp (*Laminaria digitata*) gametophyte growth and gametogenesis: effects of photoperiod and culture media. J. App. Phycol. 29:1–10.

[jpy12976-bib-0038] Reed, D. C. 1990 The effects of variable settlement and early competition on patterns of kelp recruitment. Ecology 71:776–87.

[jpy12976-bib-0039] Reed, D. C. , Neushul, M. & Ebeling, A. W. 1991 Role of settlement density on gametophyte growth and reproduction in the kelps *Pterygophora californica* and *Macrocystis pyrifera* (Phaeophyceae). J. Phycol. 27:361–6.

[jpy12976-bib-0040] Suggett, D. J. , Moore, C. M. , Hickman, A. & Geider, R. J. 2009 Interpretation of fast repetition rate (FRR) fluorescence: Signatures of phytoplankton community structure versus physiological state. Mar. Ecol. Prog. Ser. 376:1–19.

[jpy12976-bib-0041] Wiencke, C. , Roleda, M. Y. , Gruber, A. , Clayton, M. N. & Bischof, K. 2006 Susceptibility of zoospores to UV radiation determines upper depth distribution limit of Arctic kelps: evidence through field experiments. J. Ecol. 94:455–63.

[jpy12976-bib-0503] van den Burg, W. K. S. , van Duijn, A. , Bartelings, H. , van Krimpen, M. M. & Poelman, M. 2016 “The economic feasibility of seaweed production in the North Sea”. Aquaculture Economics Management 20(3):235–52. 10.1080/13657305.2016.1177859

[jpy12976-bib-0042] Zhao, X. B. , Pang, S. J. , Liu, F. , Shan, T. F. , Li, J. , Gao, S. Q. & Kim, H. G. 2016 Intraspecific crossing of *Saccharina japonica* using distantly related unialgal gametophytes benefits kelp farming by improving blade quality and productivity at Sanggou Bay, China. J. App. Phycol. 28:449–55.

