# Supplementary figures and images for: How light and biomass density influence the reproduction of delayed Saccharina latissima gametophytes (Phaeophyceae)
Source: J Phycol. 2020 Feb 28;56(3):709–18. doi: 10.1111/jpy.12976 (PMC7318604; doi:10.1111/jpy.12976)

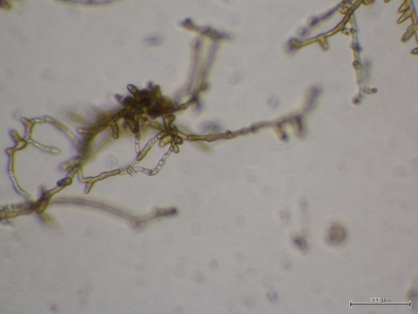

Supplement: Supplementary file 1 — Figure S1. Calibration curve between the chlorophyll a concentration (mg Chl · m−3), and Saccharina latissima gametophyte dry weight per mL (mg DW · mL−1). Gametophyte dry weights are extrapolations from 60 mL cultures, whose [Chl] concentration were measured using a FRRF fluorometer. The linear regression and correlation coefficient were y = 7E‐05x − 9E‐05 and 0.975 respectively. [file JPY-56-709-s001.tif]

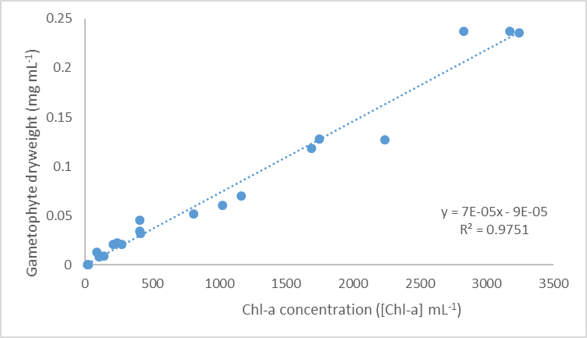

Supplement: Supplementary file 2 — Figure S2. The interaction between light intensity (μmol photons · m−2 · s−1 ) and the light quality (white, blue, red, and yellow) on Saccharina latissima gametophyte biomass (mg DW · mL−1) of cultures starting with the Initial Gametophyte Density of 0.01 mg DW · mL−1. Biomass was measured on day 21 and the error bars are ± SE, n = 36. [file JPY-56-709-s002.tif]

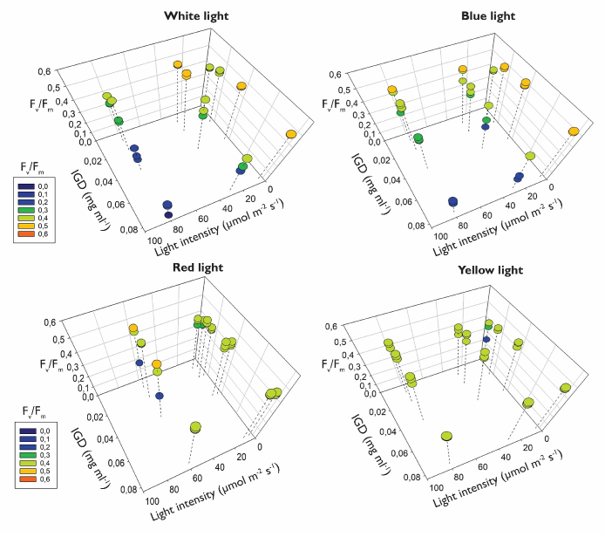

Supplement: Supplementary file 3 — Figure S3. The 3D scatterplot showing the interaction between the Fv/Fm, the IGD (mg · mL−1), and light intensity (μmol photons · m−2 · s−1) of Saccharina latissima gametophyte cultures grown under four different light qualities. The color of the dots correspond with the legend (white, blue, red, and yellow), thus corresponding with the F v/F m value of the sample n = 144. [file JPY-56-709-s003.tif]

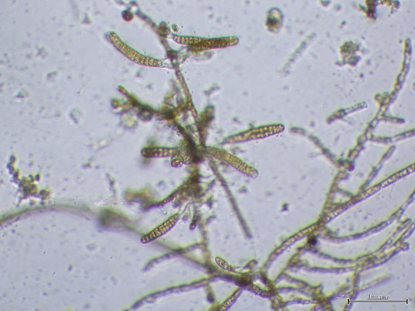

Supplement: Supplementary file 4 — Figure S4. Scatterplots depicting the Saccharina latissima gametophyte biomass measured on day 21 (y‐axis) under different levels of Photosynthetically Usable Radiation (μmol photons · m−2 s−1). Four different light qualities (white, blue, red, and yellow) were used to grow out gametophyte cultures starting with four different Initial Gametophyte Densities (0.01, 0.02, 0.04, and 0.08 mg DW · mL−1). Values are “as is,” n = 36. [file JPY-56-709-s004.tif]

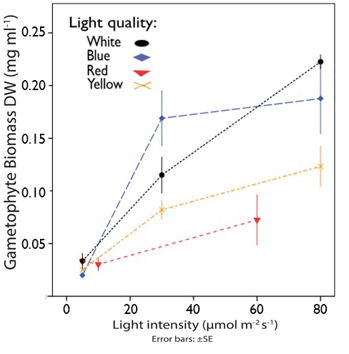

Supplement: Supplementary file 5 — Figure S5. A photo of the starting culture in a well plate (IGD = 0.01 mg DW · mL−1). [file JPY-56-709-s005.tif]

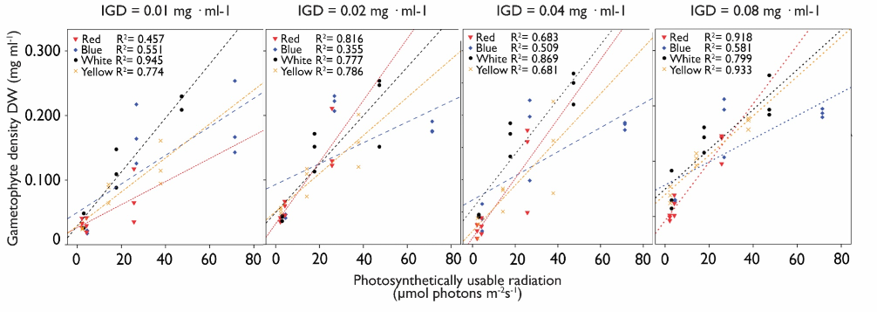

Supplement: Supplementary file 6 — Figure S6. A photo of a culture on day 21 (IGD = 0.01 mg DW · mL−1, 30 μmol · m−2 · s−1, white light). Sporophytes only formed on the bottom with gametophyte biomass being a bit blurry since it grew upward toward the light, out of focus. [file JPY-56-709-s006.tif]

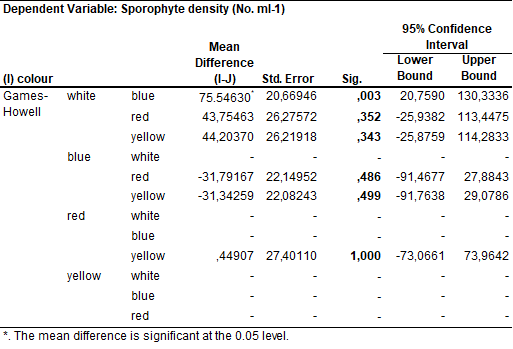

Supplement: Supplementary file 7 — Table S1. Predictors for the regression describing the correlation of the IGD and PUR on the reproduction of Saccharina latissma gametophytes in Figure 3 (n = 102). Included is the R 2 of the primary (PUR) and secondary (IGD) predictor combined. [file JPY-56-709-s007.tif]

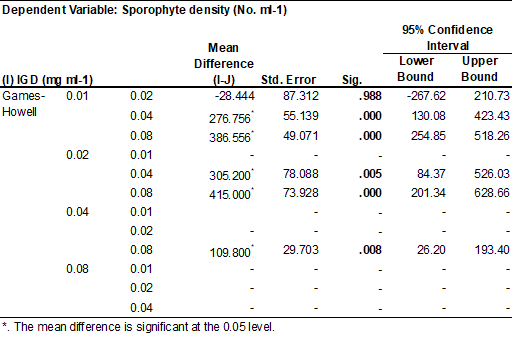

Supplement: Supplementary file 8 — Table S2. Predictors for the regression describing the correlation of PUR and the reproduction of Saccharina latissima gametophytes in Figure 2, using an IGD of 0.01 mg · mL−1. [file JPY-56-709-s008.tif]

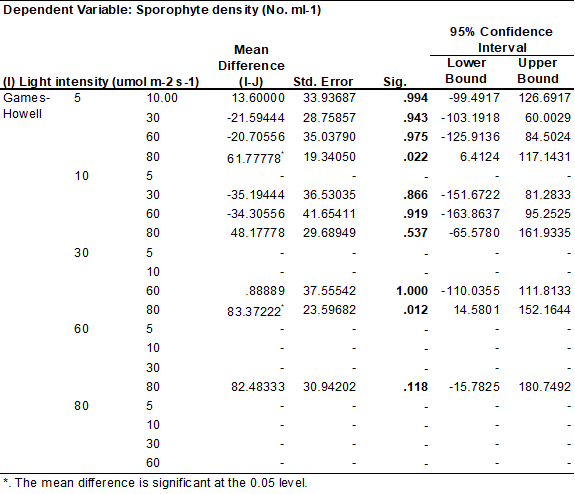

Supplement: Supplementary file 9 — Table S3. Games–Howell post hoc analysis for the influence of light quality on gametogenesis after we found significant differences using the robust test of variance. The mean difference is significant at P < 0.05. [file JPY-56-709-s009.tif]

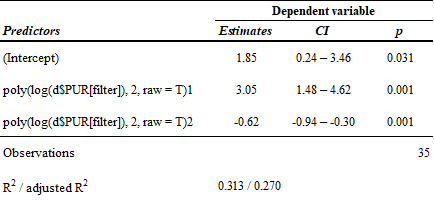

Supplement: Supplementary file 10 — Table S4. Games–Howell post hoc analysis for the influence of the IGD on gametogenesis after we found significant differences using the robust test of variance. The mean difference is significant at P < 0.05. [file JPY-56-709-s010.tif]

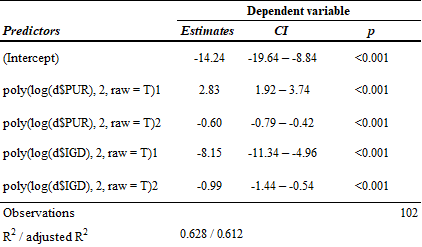

Supplement: Supplementary file 11 — Table S5. Games–Howell post hoc analysis for the influence of light intensity on gametogenesis after we found significant differences using the robust test of variance. The mean difference is significant at P < 0.05. [file JPY-56-709-s011.tif]

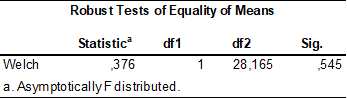

Supplement: Supplementary file 12 — Table S6. Robust test of variance for the effects of nutrients on the gametogenesis of Saccharina latissima gametophytes (Fig. 4; Welch and Brown‐Forsythe), after not passing the test of homogeneity of variances. [file JPY-56-709-s012.tif]

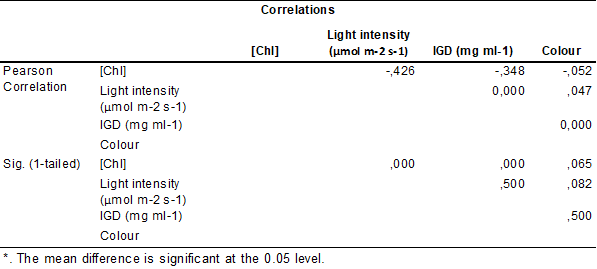

Supplement: Supplementary file 13 — Table S7. Stepwise linear regression for the correlation between the gametophyte biomass on day 21 (mg DW · mL−1), the IGD, light intensity, and light quality (n = 144). [file JPY-56-709-s013.tif]

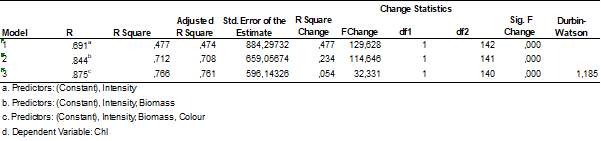

Supplement: Supplementary file 14 — Table S8. Predictors that significantly influence gametophyte growth. Included is the R 2 of the primary (IGD) and secondary predictor (light intensity) combined. [file JPY-56-709-s014.tif]

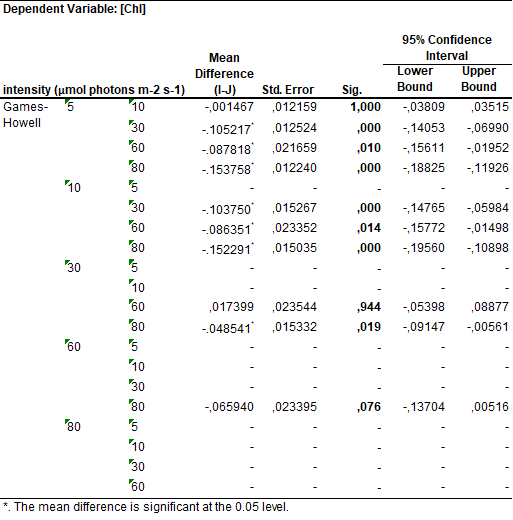

Supplement: Supplementary file 15 — Table S9. Games–Howell post hoc analysis for the influence of light intensity on the growth of gametophyte biomass (chlorophyll‐a concentration) on day 21 after we found significant differences using the robust test of variance. The mean difference is significant at P < 0.05. [file JPY-56-709-s015.tif]

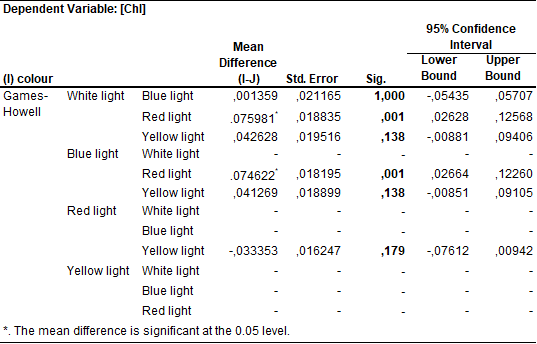

Supplement: Supplementary file 16 — Table S10. Scheffe post hoc analysis for the influence of the different IGDs on the growth of gametophyte biomass (chlorophyll‐a concentration) on day 21 after we found significant differences using a one‐way ANOVA. The mean difference is significant at P < 0.05. [file JPY-56-709-s016.tif]
